# Supplementary material for: Rapid turnover of life-cycle-related genes in the brown algae
Source: Genome Biol. 2019 Feb 14;20:35. doi: 10.1186/s13059-019-1630-6 (PMC6374913; doi:10.1186/s13059-019-1630-6)
Supplement: Supplementary file 3 — Protein alignments, corresponding Gblocks html file and CDS sequences for the lineage conserved gametophyte-biased genes. (ZIP 2109 kb) [file 13059_2019_1630_MOESM3_ESM.zip › Con_GA_alignments/Ec-28_001470.aa_ali.fasta-gb.htm]

Ec-28\_001470.aa.fasta.trans.aa\_ali.fasta


## Gblocks 0.91b Results

Processed file: **Ec-28\_001470.aa.fasta.trans.aa\_ali.fasta**  
Number of sequences: **2**  
Alignment assumed to be: **Protein**  
New number of positions: **183** (selected positions are underlined in blue)

```
                         10        20        30        40        50        60
                 =========+=========+=========+=========+=========+=========+
Ec-28_001470     ----------MRRAIGEHS-----------ADGKLL-EEMAYTLNAPMEDGTT-------
MSTRG.57035.1    SYNTKHGFSKVTSCTGEFFQNLYDYARKGCTDGILTSRKMSQAVDGRSAGSTTLKETAYW
                                                                             


                         70        80        90       100       110       120
                 =========+=========+=========+=========+=========+=========+
Ec-28_001470     -AEFARVQITPTRGGRGTLLAFAQEWKTGALNNSHLLLHSKKPDVVNNAGGFGEGFKVAA
MSTRG.57035.1    LNEDARVQITPTDQGRRTRLAFAQLWD-GALNNSHLLLHSKKGDGRGNVGGYGEGFKVAA
                   ########################  ################################


                        130       140       150       160       170       180
                 =========+=========+=========+=========+=========+=========+
Ec-28_001470     NSLLARFPNSSAEPCNILMVMNGRTWRFCYRNFETDGTVASSEKIDQLVVDEYST-KVYG
MSTRG.57035.1    NSLLGQFAACSVEPCQVWMVMNSRTWRFCYKEFKPDYLTTSSEKIEQLVVEEYKTQKALG
                 ####################################################### ####


                        190       200       210       220       230       240
                 =========+=========+=========+=========+=========+=========+
Ec-28_001470     GMVVFVEIPMSATSIERIWNPEHYVPTMSLATWTPLLPPGASPVMSYVLFLEDSNAPSKF
MSTRG.57035.1    GMVVFIDIPMAPTSLNKFWNPNHYIPTTPLADWIPLLPAGELPVMSYVRFLDDSNAPSQV
                 ############################################################


                        250       260       270       280       290       300
                 =========+=========+=========+=========+=========+=========+
Ec-28_001470     FQLGIYVGSVDNSKGAFFNEGGSVNIGRDRSKTSDVLSNIRTAIAKRVKLLPAGTRDDAV
MSTRG.57035.1    FQLGIYVGEMH-------------------------------------------------
                 ########                                                    


                        310       320       330       340       350       360
                 =========+=========+=========+=========+=========+=========+
Ec-28_001470     MERLMVNPDALGQEPRWYGSRVLYKSVVSYVRSRDRVPDTTEIFLVGAEIAREVTERLRG
MSTRG.57035.1    ------------------GSKV--------------------------------------
                                                                             


                        370       380       390       400       410       420
                 =========+=========+=========+=========+=========+=========+
Ec-28_001470     AQVNAHVVSAEKELWTYESNEQVSKVLMTKTFAELSDSQRQHFQVELQILATISPSRCVW
MSTRG.57035.1    ------------------------------------------------------------
                                                                             


                        430       440       450       460       470       480
                 =========+=========+=========+=========+=========+=========+
Ec-28_001470     VLGKWPGTYFQAYMGQSKRYFHDTVSNRYLVPAVLLKDQAFWPGLCMDAWTLSKLDPYIL
MSTRG.57035.1    -------------------------------------------GAC--------------
                                                                             


                        490       500       510       520       530       540
                 =========+=========+=========+=========+=========+=========+
Ec-28_001470     MQRVGSDHSILHDPNRVRTMVTKAREQAASGNGLLVGADSNRYASPEGAVEPPPPRAIFG
MSTRG.57035.1    ------------------------------------------------------------
                                                                             


                        550       560       570       580       590       600
                 =========+=========+=========+=========+=========+=========+
Ec-28_001470     GNSDRPPNSDSGGSAVYPPNGQPPSVGGPAVRPRRGMADQPQWFPPPTLKDPSGLSIGGQ
MSTRG.57035.1    ------------------------------------------------------------
                                                                             


                        610       620       630       640       650       660
                 =========+=========+=========+=========+=========+=========+
Ec-28_001470     SGRSNDIVDSVDEDEETRLKPGPLRCDLGVELFVAHGVSDLEASRFYDRFMTTPGVIDAL
MSTRG.57035.1    ----------------------------------------LEAS----------------
                                                                             


                        670
                 =========+====
Ec-28_001470     HTIFLTARSFHFSX
MSTRG.57035.1    --------------
```

```
Parameters used
Minimum Number Of Sequences For A Conserved Position: 2
Minimum Number Of Sequences For A Flanking Position: 2
Maximum Number Of Contiguous Nonconserved Positions: 8
Minimum Length Of A Block: 10
Allowed Gap Positions: None
Use Similarity Matrices: Yes
```

```
Flank positions of the 3 selected block(s)
Flanks: [63  86]  [89  175]  [177  248]  

New number of positions in Ec-28_001470.aa.fasta.trans.aa_ali.fasta-gb:  183  (27% of the original 674 positions)
```
